# Behavioral Outcomes of Children with Same-Sex Parents in The Netherlands

**DOI:** 10.3390/ijerph19105922

**Published:** 2022-05-13

**Authors:** Deni Mazrekaj, Mirjam M. Fischer, Henny M. W. Bos

**Affiliations:** 1 Department of Sociology, Utrecht University, Padualaan 14, 3584 CH Utrecht, The Netherlands; 2 Nuffield College, University of Oxford, New Road, Oxford OX1 1NF, UK; 3 Leuven Economics of Education Research (LEER), KU Leuven, Naamsestraat 69, 3000 Leuven, Belgium; 4 Institute of Sociology und Social Psychology, University of Cologne, Albertus-Magnus-Platz, D-50923 Cologne, Germany; m.fischer@uni-koeln.de; 5 Research Institute Child Development and Education, Faculty of Social and Behavioral Sciences, University of Amsterdam, Nieuwe Achtergracht 127, 1001 NG Amsterdam, The Netherlands; h.m.w.bos@uva.nl

**Keywords:** same-sex parents, behavioral outcomes, family system theory, minority stress theory, probability sample, coarsened exact matching

## Abstract

Same-sex parents face substantial stressors due to their sexual orientation, such as experiences of prejudice and prohibitive legal environments. This added stress is likely to lead to reduced physical and mental health in same-sex parents that, in turn, may translate into problematic behavioral outcomes in their children. To date, there are only a few nationally representative studies that investigate the well-being of children with same-sex parents. The current study takes a closer look at children’s behavioral outcomes, reported by a parent, using an adapted version of the emotional, conduct, hyperactivity, pro-social, and peer problems subscales of the Strengths and Difficulties Questionnaire (SDQ). We take advantage of unique data from the Netherlands based on a probability sample from population registers, whereby findings can be inferred to same-sex and different-sex parent households with parents between the ages of 30 and 65, and with children between the ages of 6 and 16 years (62 children with same-sex, and 72 children with different-sex parents). The findings obtained by coarsened exact matching suggest no significant disadvantages for children with same-sex parents compared to different-sex parents. We contextualize these findings in their wider cultural context, and recommend a renewed focus in future research away from deficit-driven comparisons.

## 1. Introduction

According to family system theory, families consist of interdependent subsystems. As such, stress and emotions experienced in one subsystem (e.g., parents) are inextricably linked to behaviors and feelings in other family subsystems (e.g., children) [1,2]. Such a spillover effect may explain why parents’ experience of stress influences their children’s psychological outcomes [3]. After all, high levels of parental stress contribute to an increased risk of parental psychological problems [4] and, in turn, to children’s psychological and behavioral problems [5,6].

Same-sex parents face stress due to their sexual orientation, such as experiences of prejudice, negative feedback from friends and family, and a prohibitive legal environment [7,8,9,10,11]. The literature on minority stress theory continually shows adverse mental health outcomes for sexual minorities, resulting from the stress of navigating heterosexist societies [12,13,14]. Same-sex parents anticipate rejection not only of themselves, but they expect the rejection of their children, which adds stress unique to same-sex parents to general stress experienced by all parents [9,15,16,17,18]. Combining insights from family system theory and minority stress theory, one can assume that children growing up in same-sex parent families may experience more psychological problems than children growing up in different-sex parent families due to excessive stress on the family system as a whole.

In contrast to this theoretical prediction, the body of empirical literature examining potential differences between children raised by same-sex or different-sex parents finds small or no differences in terms of behavioral problems [19], and only a few studies find small disadvantages in terms of emotional adjustment and schooling outcomes [20]. Regardless of the outcome, previous studies have been criticized for methodological shortcomings, in particular the use of nonprobability samples (i.e., recruited at lesbian and gay parenting groups or fertility clinics). It is possible that parents who are recruited through lesbian and gay parenting groups want to show that they are good parents. Therefore, their answers might be biased on their children’s behavior and adjustment. Another critique of these studies is their limited statistical power to detect significant differences [16]. Therefore, to draw more general conclusions, it is advisable to compare the well-being of children in same-sex parent and different-sex parent families in national probability samples rather than in surveys of specific groups.

To overcome selection bias in community samples, the field has turned to research based on household surveys of the general population. In some of these surveys, it is possible to identify same-sex and different-sex couples. Same-sex couples who live together can be identified in household grids if the gender and the nature of the relationship between household members are recorded [21]. Findings based on this work draw similar conclusions to the nonprobability literature, namely small or no differences in terms of health, psychological and behavioral adjustment, delinquency, and school outcomes between children raised by same-sex and different-sex parents [22,23,24,25,26,27,28].

Though the household grid approach allows far more general conclusions about same-sex parent families than studies using nonprobability surveys, these studies face other methodological challenges. Surveys, which are not designed explicitly with same-sex relationships in mind, are susceptible to errors due to careless heterosexist assumptions during data collection and processing [21]. Moreover, same-sex relationships tend to make up a numerically small group relative to different-sex couples, leading to low statistical power (i.e., true differences between the groups may not be detected [29]). Moreover, otherwise negligible misclassifications based on the household grid—e.g., random mistakes in either the respondent’s gender, the household member’s gender, or the nature of the relationship between respondent and the household member—become substantial sources of error. Suppose different-sex parents are misclassified into the group of same-sex parent families, the group size is inflated considerably, which may introduce bias into the substantive conclusions drawn based on these data.

Two recent studies have taken advantage of population registers in the Netherlands to examine schooling outcomes of all children with same-sex and different-sex parents, circumventing any sampling bias and power issues [30]. The authors find that children raised by same-sex parents from birth outperform children raised in different-sex families in primary and secondary education. Specifically, children with same-sex parents further have higher standardized tests scores, high school graduation rates, and college enrollment. In Sweden, this finding can be replicated for boys, whereas girls’ school performance does not differ across same-sex and different-sex parents [31]. This evidence is based on exceptionally sound methodology. Yet, the range of substantive topics, which can be addressed with data from population registers, is limited to socio-demographic and a handful socio-economic outcomes (e.g., family constellation, schooling, and parental income).

The current study expands on recent efforts to overcome methodological challenges that commonly plague this type of research. We use survey data based on a probability sample from population registers to examine children’s behavioral outcomes, reported by a parent, using an adapted version of the emotional, conduct, hyperactivity, pro-social, and peer problems subscales of the Strengths and Difficulties Questionnaire (SDQ). Thus, our goal is to find out whether children with same-sex parents score differently on the behavioral subscales than children with different-sex parents. The data used in this study are unique in that they are probability-based on a sampling frame from Dutch population registers [32]. The Netherlands is a country where same-sex families are widely recognized, with extensive legal and cultural support provided to same-sex parents. The Netherlands was the first country to introduce same-sex marriage in 2001 along with adoption rights for same-sex couples (registered partnerships for both same- and different-sex couples were already possible since 1998). In 2014, the parental law was updated so that women in same-sex relationships could obtain parental rights to a child born by their partner without having to go through second-parent adoption.

## 2. Materials and Methods

### 2.1. Sample Description

This study uses survey data based on a probability sample among people between the ages of 30 and 65, who live with a same-sex or different-sex partner in the Netherlands, regardless of their marital status [33]. In the first step, municipalities were selected by a stratified sample across three geographical regions and three levels of urbanization (response rate 61%, realized *n* = 20 municipalities). In the second step, a sampling frame for same- and different-sex couples was created by means of an approximation strategy. Households in which two persons between the ages of 30 and 65 live, who are not siblings or parent and child to each other, make up the frame of same- and different-sex couples. A third class of households, same-sex couples with children, was created by adding the condition that a child under the age of 18 had to live at the same address, regardless of the legal parental status. This condition was not used among different-sex couples, as the prevalence of households with children is high enough to obtain sufficient parent households without an oversample. Both the couple and the parental status was later confirmed in the survey. Estimation accuracy, i.e., the percentage of households confirming their relationship status as it was estimated in the approximation, was at 90%. Local authorities then drew random samples from their population registers within these three types of households, with an oversample of same-sex couples with and without children by a factor of three. As the total number of same-sex households with and without children is known in the registers, weights could be calculated to correct for the stratified sample and the oversample using the distributions from the population registers.

Results can be inferred from the population of two-parent families with children between the ages of 6 and 16, and their parents between the ages of 30 and 65 in the Netherlands. Same-sex couples with and without children were oversampled to obtain sufficient observations to detect statistically significant group differences. The sample was stratified by geographical region and level of urbanization to ensure the meaningful inclusion of same-sex couples in rural communities. The risk of misclassification is reduced considerably due to the identification in the population registers and the self-identification as same- or different-sex couples in the subsequent survey.

The surveys were web-based, and participants gave informed consent on the start page of the survey before participating. Response was highest among same-sex couples with children (34%), followed by same-sex couples without children (27%), and mixed-sex couples with or without children (20%). After removing households in which partners gave conflicting or incomplete answers about their couple status (*n* = 13), and households where the two adults were not a couple (*n* = 42), the sample contains a total of 1353 valid individual cases in 880 households. The study was approved by the Amsterdam Institute for Social Science Research Ethical Advisory Board (protocol code 2015-AISSR-6327 on 21 March 2016).

### 2.2. Measures

Our outcome of interest is the children’s total problem behavior, which was measured with an adapted version of the Strengths and Difficulties Questionnaire—SDQ [34]. To compute the total problem behavior score, we first computed five SDQ subscales: the emotional problems scale, the conduct problems scale, the hyperactivity scale, the anti-social scale, and the peer problems scale. Each scale includes several statements about the child. The emotional problems scale included three statements (e.g., “my child is often unhappy, down”). The conduct problems scale included two statements (e.g., “my child often has temper tantrums, hot tempers”). The hyperactivity scale also consisted of two statements (e.g., “my child is restless, overly active, cannot sit still for long”). The anti-social scale included five statements (e.g., “my child does not feel at ease in social situations, such as a party, school, playing with others”), and the peer problems scale included two statements (e.g., “my child gets picked on or bullied by other children”). For each statement, the parents could choose among four answers: “not true”, “somewhat true”, “certainly true”, and “I don’t know”. In line with the SDQ manual, we gave a score of 0 if a negative statement “was not true”, a score of 1 if it was “somewhat true”, and a score of 2 if it was “certainly true” ([35], https://www.sdqinfo.org/ accessed on 24 February 2022). The reverse coding was used for positive statements, e.g., “is generally liked by other children”. We coded the answer “I don’t know” as missing. Then, a composite score per scale was obtained by summing up the individual scores per statement, with a higher score meaning more behavioral problems. If one of the statement scores was missing, the score on the entire scale was counted as missing. The outcome of interest, children’s total problem behavior, was obtained as a sum of the scores on the five behavioral scales. By construction, this score can take on the values of 0 to 28 (two multiplied by 14 statements), with a higher score indicating that the child has more behavioral difficulties. We also computed two additional scales that are commonly analyzed in the literature: the externalizing and the internalizing problem behavior scores. The externalizing problem behavior score was calculated as the sum of the conduct problems scale and the hyperactivity scale, and the internalizing problem behavior score as the sum of the emotional problems scale and the peer problems scale.

The independent variable of interest is an indicator given a value of 1 if the child has same-sex parents and 0 if the child has different-sex parents. To construct this variable, we used the answers to two questions. First, respondents were asked about their own gender. Then, the respondents were asked about the gender of their partner. In both cases, the respondents could choose between “male” or “female”. Thus, we consider a child to have same-sex parents if the respondents report their gender and their partner’s gender to be the same (both female or both male). Consequently, a child is considered to have different-sex parents if the respondent’s gender is female and the partner’s gender is male or vice-versa.

At the child level, we control for gender of the child by including an indicator given a value of 1 if the child is female and 0 if the child is male. We also control for age of the child by including a continuous age variable. At the parental level, we control for socioeconomic status of the parent by including respondents’ education and income. Education is given a value of 1 if the respondent obtained a higher education diploma (Bachelor, Master, or Ph.D.) and a value of 0 if the respondent either only had a high school diploma or was a high school dropout. Income includes the respondent’s personal monthly net income from work in euros, and is constructed as a categorical variable that includes three categories: low (less than 1499 euros per month), middle (from 1500 to 2999 euros per month), and high (more than 2999 euros per month). Finally, we control for three family variables: marital status (0 is not married, 1 is married), whether the child is born outside the current relationship (0 is no, 1 is yes), and a continuous variable for the total number of children living in the household. All control variables have been previously used in the literature on children’s outcomes in same-sex families (see, for instance [36]).

### 2.3. Sample Restrictions

To study the behavioral outcomes of children with same-sex parents, we restricted the sample in three ways. We started off with a sample of 1353 valid individual cases in 880 households. As we are interested in children’s outcomes, we removed respondents without children, resulting in 603 respondents in 412 households. Then, we removed respondents whose children were either younger than 6 or older than 16, or whose children do not live with the respondent. This is because the statements to compute the scales were about children between 6 and 16 years old that still live with the respondent. Thus, if the child was outside this age range or did not live with the respondent, the statements to compute the scales were missing and we could not analyze the children’s behavioral outcomes. This restriction reduces the sample to 341 respondents in 234 households. Further, some respondents lived together in the same household, and were therefore questioned about the same child. To avoid double counting, we followed simple transparent rules to decide which respondent to keep based on assumptions on which parent spends most time in the household. Namely, we first selected women, given that studies show that women still conduct most of the household labor and childcare [37,38]. Then, we selected unemployment status and hours worked, assuming that parents that are unemployed or work less also spend more time in the household with children. This resulted in a sample of 241 unique respondents in 234 households. Finally, we removed missing values on the outcomes, the independent variable, and the covariates, resulting in a total sample of 186 children, among which 74 children have same-sex parents and 112 children have different-sex parents.

### 2.4. Statistical Analysis

We compared children with same-sex parents to children with different-sex parents using Coarsened Exact Matching (CEM). CEM is particularly useful in reducing the imbalances in observed characteristics between two groups, and reducing model imbalances [39,40] that may lead to treatment effect bias [41]. CEM achieves this by matching each child with same-sex parents to one or several children with different-sex parents who have either exactly the same observed characteristics (exact matching) or very similar observed characteristics based on narrow categories (coarsened exact matching). Iacus, King, and Porro (2012) found that coarsened exact matching outperforms both linear regression estimated by OLS and the often used Propensity Score Matching (PSM) in estimating causal effects [39]. Therefore, we believe CEM is particularly useful for this analysis. Nonetheless, as we cannot control for unobserved factors, our results are correlational and not causal. To perform the matching, we searched for an exact match on all covariates, except for the number of children in the household, which was coarsened to include three categories (one child, two children, three or more children), and age of the child, which was kept continuous. We kept age continuous given that there was no theoretical basis for coarsening. Given that there was no perfect match for some of the variables, our final sample includes 134 children, among which 62 children have same-sex parents. G*power version 3.1.9.1 (Heinrich Heine University Düsseldorf, Düsseldorf, Germany) was used to conduct post hoc power analyses with α = 0.05 [42]. Analyses revealed very high power (1−β error probability = 0.977) to detect moderate effect sizes (f^2^ = 0.10).

## 3. Results

In this section, we first present descriptive statistics on children with same-sex and different-sex parents, respectively. Then, we estimate the relationship between having same-sex parents and the total problem behavior score. We also present the results for the externalizing and internalizing behavioral problems scores separately. Finally, we estimate the relationship between having same-sex parents and each of the five behavioral scales: the emotional problems scale, the conduct problems scale, the hyperactivity scale, the anti-social scale, and the peer problems scale.

### 3.1. Descriptive Statistics

Table 1 shows that, before coarsened exact matching, children with same-sex parents are significantly more likely to have parents who have finished higher education. Moreover, children with same-sex parents grew up with less other children in the household. These two factors may suggest that children with same-sex parents tend to have a socioeconomic advantage over children with different-sex parents. However, Table 1 also shows that children with same-sex parents are 16.8 percentage points more likely to be born outside of the respondent’s current relationship, suggesting that children with same-sex parents are significantly more likely to have experienced parental separation. This is consistent with other studies that used Dutch administrative population data [43].

### 3.2. Relationship between Having Same-Sex Parents and Behavioral Outcomes

**Figure 1:**
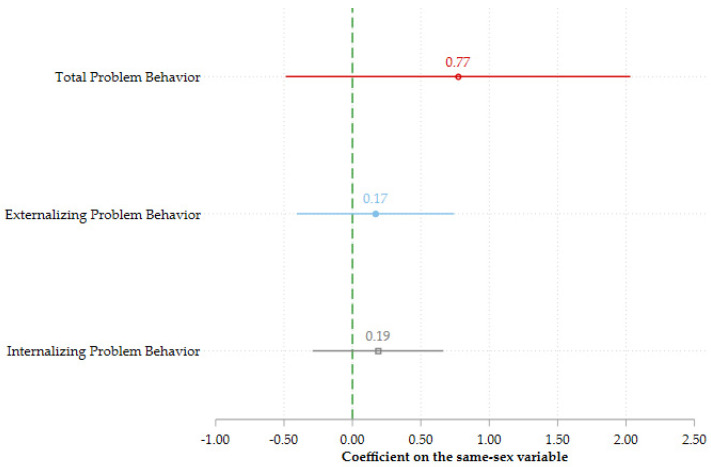
Relationship between having same-sex parents and difficulties, externalizing, and internalizing scores. The bars represent the 95% confidence intervals. All models have been estimated using coarsened exact matching on the gender and age of the child, marriage status of the respondent, education and income of the respondent, whether the child was born outside of the respondent’s current relationship, and the number of children in the household. The sample includes 62 children with same-sex parents and 72 parents with different-sex parents.

Figure 1 compares children with same-sex parents to children with different-sex parents on the total problem behavior score, and the externalizing and internalizing problem behavior scores. All models have been estimated using coarsened exact matching on gender and age of the child, marriage status of the respondent, education and income of the respondent, whether the child was born outside of the respondent’s current relationship, and the number of children in the household. Although the coefficients are positive, the confidence intervals are very wide and include zero for both total problem behavior (estimate = 0.772, se = 0.637, *p* = 0.227, 95% CI (−0.487, 2.032)), as well as the subscales for externalizing (estimate = 0.168, se = 0.291, *p* = 0.565, 95% CI (−0.408, 0.743)) and internalizing problem behavior (estimate = 0.187, se = 0.440, *p* = 0.004, 95% CI (−0.291, 0.665)). Thus, we do not observe any significant differences on any of the scores. This suggests that children with same-sex parents do not experience more behavioral difficulties, externalizing or internalizing, than children with different-sex parents.

**Figure 2:**
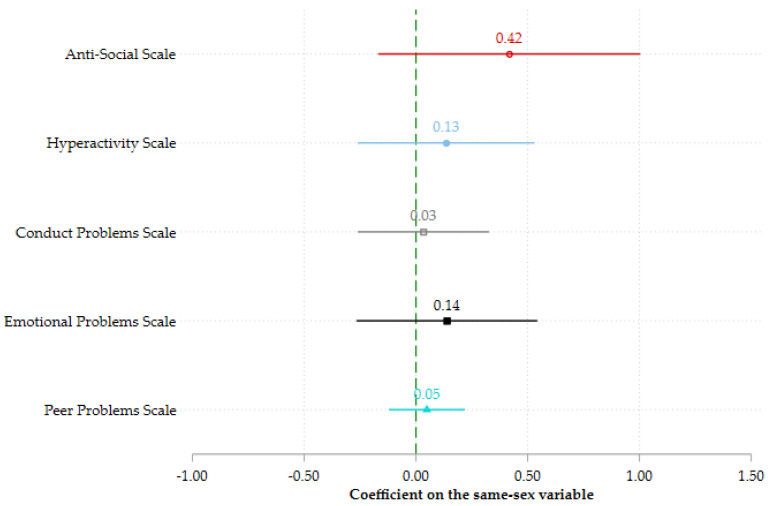
Relationship between having same-sex parents and behavioral scales. The bars represent the 95% confidence intervals. All models have been estimated using coarsened exact matching on gender and age of the child, marriage status of the respondent, education and income of the respondent, whether the child was born outside of the respondent’s current relationship, and the number of children in the household. The sample includes 62 children with same-sex parents and 72 parents with different-sex parents.

In Figure 2, we dig deeper into behavioral difficulties, and compare children with same-sex parents to children with different-sex parents on each of the five behavioral scales: the emotional problems scale (estimate = 0.138, se = 0.204, *p* = 0.500, 95% CI (−0.266, 0.543)), the conduct problems scale (estimate = 0.033, se = 0.148, *p* = 0.825, 95% CI (−0.260, 0.326)), the hyperactivity scale (estimate = 0.135, se = 0.200, *p* = 0.501, 95% CI (−0.261, 0.531)), the anti-social scale (estimate = 0.417, se = 0.296, *p* = 0.162, 95% CI (−0.169, 1.004)), and the peer problems scale (estimate = 0.049, se = 0.086, *p* = 0.571, 95% CI (−0.121, 0.219)). Once again, the coefficients are positive, but none of the coefficients are significantly different from zero. Moreover, all coefficients except the anti-social scale coefficient are very close to zero. Thus, the more detailed behavioral scale results confirm that children with same-sex parents do not experience more behavioral difficulties than children with different-sex parents.

## 4. Discussion

The current study used unique probability survey data of children with same- and different-sex parents in the Netherlands to examine children’s behavioral outcomes. Specifically, we studied the emotional, conduct, hyperactivity, pro-social, and peer problems subscales of the SDQ as reported by a parent. Our results show that children in both family types show similar levels of behavioral adjustment, and that no statistically significant differences between children with same- and different-sex parents can be found. These findings are in line with the overwhelming majority of prior research in this field. Considering the mounting evidence on minority stress experienced by sexual minorities in general, and by sexual minority parents in particular, this remains somewhat of a puzzle. Why does the disproportionally high prevalence of stress-related psychological morbidities, such as depression, anxiety, and other mental disorders, among sexual minority people not translate into negative outcomes in their offspring?

Part of the answer may be that same-sex parents prepare their children for the confrontation with the heteronormative society and adverse reactions to their family situation to cope with this, so that it does not influence their behavior or well-being [44,45,46,47]. Another possible explanation for our no-differences finding may be the relatively supportive legal and social environment for same-sex parents and their children in the Netherlands. Even though there continues to be ample room for improvement, for example, with equal access to in vitro fertilization for female same-sex couples, gestational surrogacy for male–male couples, and a legal parenthood status for all involved parents in case of international multiple parenthood (e.g., a couple of two men who are parenting one or more children together with a couple of two women while living in two different households), the Netherlands can be considered a legal frontrunner for same-sex families. Nonetheless, sexual minorities continue to face social rejection, stigma, and discrimination in the Netherlands and elsewhere [46,48,49]. Recent evidence shows that same-sex couples in the Netherlands face social exclusion from their families-of-origin more often than different-sex couples do [50]. Structural differences in their social networks point towards the observation that latent heterosexism continues to affect sexual minority couples and families. Research on the consequences of legal support for same-sex couples and families show that formal support structures are a crucial resource of resilience against heterosexist barriers to sexual minority family functioning, in general [51,52]. In light of this, legal support must continue to be strengthened to ensure the well-being of children with same-sex parents and their families.

Our findings suggest that there is considerable resilience within the family systems of minority families in order for mental health disparities not to get passed down to children. We do know that individual resilience factors to buffer against adverse mental health outcomes in sexual minorities include having a strong support network and ties to a community of other sexual minority people and families [53,54]. Yet, neither the resilience of children with same-sex parents nor the link between parental mental health and children’s behavioral outcomes in sexual minority families have received much scientific attention (for an exception, see [55]). As a result, little is known about the mechanisms underlying the resilience of their family systems. A better understanding of both stressors and sources of resilience for children with same-sex parents and their families is needed to facilitate targeted support to those who need it. Mean comparisons such as ours, and that of the majority of the quantitative research in the field, simplify the heterogeneity within the groups of children. Both comparison groups include children and families who do need additional support. Knowing that same-sex parents are at higher risk for adverse mental health outcomes, the link between parental and children’s adjustment is one obvious target for further support for sexual minority families.

For many decades now, the well-being of children with same-sex parents has been under scrutiny in an effort to instrumentalize evidence of lower child well-being in the political struggles against adoption rights. It is, therefore, conceivable that the knowledge about such heightened scrutiny of their parenting and their families may give same-sex parents incentive to underreport any problems their children might have. Yet, this downward bias is unlikely to be the sole reason why no differences in adjustment between children with same-sex and different-sex parents are found over and over again. Another reason for the observed discrepancy between elevated stress and a marked health gradient for same-sex parents and their children’s behavioral adjustment may be the active compensation strategies of same-sex parents. It has been acknowledged in past research that there is a selection into parenthood, since becoming parents is associated with considerable legal, financial, and social hurdles. Same-sex couples who become parents in spite of these obstacles must have a strong desire to become parents, which is not paralleled by the average different-sex parents. In light of this, true differences in the quality of parenting are plausible.

Regardless, it is important to understand and strengthen sexual minority families legally and socially, since they should not have to have such marked resilience against the adversities their families face in heterosexist societies. To this end, avenues for future research include shifting the focus away from a deficit-driven comparative approach between children with same-sex and different-sex parents towards understanding mechanisms of stress and resilience within sexual minority families [55].

As with any study, ours comes with limitations. We only used the parental version of the SDQ that relies on self-reports by the parents. The measures are not free of idiosyncratic differences in the subjective perception of the child’s behavior and, in some cases, social desirability. This may pose a problem if the tendency to underreport problems is correlated with the family type. Yet, studies of objective outcomes such as school enrollment and performance also suggest that children with same-sex parents do equally well or better than children with different-sex parents. This lends credibility to findings based on subjective measures of child adjustment, such as the parent version of the SDQ.

Moreover, we cannot account for selection bias on the basis of individual characteristics into the survey. The weights in the UNICON data allow for a correction of the total number of same-sex and different-sex parents per cell (region × urbanization). Yet, since we do not know any further information about the socio-demographic characteristics of the couples, it is difficult to estimate to what extent the survey suffers from selective response. This is pertinent in light of the rather low response rate. Although the response rate is not optimal, it is satisfactory considering the increasing survey fatigue among the Dutch population, and the respondent burden of transitioning from physical invitation letters to web participation in the survey [32]. Steinmetz and Fischer (2019) conducted an indirect evaluation of possible selective response by using a comparable benchmark survey. They conclude that, within the limits of this indirect evaluation, there is at least no evidence of a strong selection bias.

Our present study was based on a “between-difference” approach by comparing children in same-sex parent families with children in different-sex parent families. Studies based on a between-difference approach cannot speak on the unique experiences of children who are growing up in a same-sex parent family (such as confrontation with the heteronormative society, homophobic stigmatization, how they are dealing with these experiences, and what it means for their psychological adjustment). It is only possible to get more information about these type of questions with within-difference approaches, where the focus is only on same-sex parent families [55]. Although there are no significant mean differences in well-being among children in same-sex and different-sex parent families (such as in our study) [56,57], there is heterogeneity within the groups of children. Several studies showed that children in same-sex parent families who experience homophobic stigmatization report more problem behavior [19,58]. The effect of these kind of experiences with homophobic stigmatization earlier in life (for example, during adolescence) can often be traced later in life during (emerging) adulthood [59,60]. However, there are also protective factors against adverse effects on children [45,46,61]. An example of such a protective factor is when the mothers create an environment for the child in which the child knows and has contact with other children who have two parents of the same sex [45]. This is important information that can help same-sex parent families to prevent difficulties in the lives of their children.

## 5. Conclusions

This study has once more strengthened the body of research that suggests no structural differences among children with same-sex and different-sex parents regarding a range of behavioral and emotional outcomes. Our study has filled a gap in the field by supplementing existing evidence from community and convenience samples, large household surveys, and population data from registers with evidence from a data source, which is entirely unique in the field. Given this converging evidence of no difference, we recommend a renewed focus away from a deficit-driven comparative approach toward understanding stress and resilience factors unique to sexual minority families.

## Figures and Tables

**Table 1:** Descriptive statistics before coarsened exact matching.

	Children with Same-Sex Parents	Children with Different-Sex Parents	Difference
Gender of child (1 is female)	0.473	0.554	−0.081
			(0.075)
Age of child	11.297	11.554	−0.256
			(0.496)
Married	0.703	0.768	−0.065
			(0.066)
Born outside relationship	0.284	0.116	0.168 ***
			(0.057)
Education (1 is higher edu.)	0.865	0.652	0.213 ***
			(0.064)
Income			
Low	0.230	0.241	−0.011
			(0.064)
Middle	0.676	0.580	0.095
			(0.073)
High	0.095	0.179	−0.084
			(0.053)
Children in household	1.932	2.313	−0.380 ***
			(0.135)

Standard errors are between parentheses. *** *p* < 0.01 (two-tailed *t*-tests).

## Data Availability

Data are available for non-commercial academic use at Fischer, M. M. (University of Amsterdam); Kalmijn, M. (University of Amsterdam); Steinmetz, S. (University of Amsterdam) (2017): Unions in Context (UNICON). DANS. https://doi.org/10.17026/dans-z5y-xa6w accessed on 3 November 2021.
